# Parallel simulation and optimization framework of supplies production processes for unconventional emergencies

**DOI:** 10.1371/journal.pone.0261771

**Published:** 2022-01-13

**Authors:** Dan Zhu, Yaoyao Wei, Hainan Huang, Tian Xie

**Affiliations:** 1 School of Economics, Management and Law, University of South China, Hengyang, Hunan Province, China; 2 School of Public Administration at Nanjing Normal University, Nanjing, Jiangsu Province, China; 3 School of Education at Central China Normal University, Wuhan, Hubei Province, China; GC Women University Sialkot, PAKISTAN

## Abstract

The outbreak of unconventional emergencies leads to a surge in demand for emergency supplies. How to effectively arrange emergency production processes and improve production efficiency is significant. The emergency manufacturing systems are typically complex systems, which are difficult to be analyzed by using physical experiments. Based on the theory of Random Service System (RSS) and Parallel Emergency Management System (PeMS), a parallel simulation and optimization framework of production processes for surging demand of emergency supplies is constructed. Under this novel framework, an artificial system model paralleling with the real scenarios is established and optimized by the parallel implementation processes. Furthermore, a concrete example of mask shortage, which occurred at Huoshenshan Hospital in the COVID-19 pandemic, verifies the feasibility of this method.

## 1 Introduction

Unconventional emergencies are characterized by heavy casualties, huge economic losses, and constraints on social development. In recent years, various unconventional emergencies, such as COVID-19 and SARS, occur frequently and tend to erupt in a short time, triggering the surging demand for emergency supplies during the initial phase of the outbreak. On the one hand, hospitals and other public places will consume large amounts of protective materials and equipment. On the other hand, the public will blindly purchase a large number of protective products due to information asymmetry and fear of unknown viruses [1]. Therefore, the surging demand makes the state of demand and supply in the conventional situation no longer balanced. If the manufacturing system fails to formulate an emergency response plan in time to meet the surging demand, it will result in more severe casualties and more significant economic losses [2].

Currently, scholars pay more and more attention to the study of emergency management of unconventional events. In response to the above-mentioned surging demand for supplies, Zeng et al. extended the modeling objects of the traditional gray simulation and prediction model from "homogeneous data" to "heterogeneous data", making the prediction results more accurate for disaster emergency supply-demand [3]. Deng et al. proposed a resource demand forecasting method based on case-based reasoning for the supply-demand problem of the emergency response of water traffic accidents [4]. Tang et al. designed and explained the whole process model of emergency supplies dispatching for large-scale emergencies from three stages of emergency supplies dispatching preparation, dispatching implementation, and dispatching evaluation [5]. Hu et al. reconstructed the urban emergency medical supplies scheduling and distribution system under public health emergencies to address the problems of unreasonable emergency medical supplies scheduling and low transfer efficiency of distribution centers in the prevention and control of the COVID-19 [6]. Zhu et al. constructed a system dynamics model for the cross-regional collaborative deployment of emergency supplies in response to the cross-regional collaborative emergency demand when major infectious diseases outbreak [7]. Xie et al. proposed a parallel simulation decision model for patient surge scenarios under unconventional public health emergencies based on the theories of parallel emergency management and discrete event system [8]. Lee et al. established a Markov decision model for the surge of patients in the emergency room caused by large-scale casualties, resulting in optimal life-saving strategies and admission control decisions [9]. Cao et al. used a multi-objective mixed-integer nonlinear programming model to formulate the relief distribution problem regarding disaster sustainability [10]. Khaled et al. designed a game theory-based model, which advocated the centralized-decentralized allocation approach to effectively enhance stock management of personal protective equipment supply in healthcare facilities during the COVID-19 outbreak [11]. Xie et al. constructed a cross-domain integration and reasoning space model to solve the problem of heterogeneity and disorder in the cross-domain integrated emergency response processes [12]. Jin et al. proposed a bipartite graph convolution neural network model to predict the emergency medical service (EMS) demand between hospitals and regions, seeking to meet EMS supply and demand [13]. Cao et al. proposed sustainable multi-period relief distribution with fuzzy supplies in response to supply shortage and inequitable distribution under large-scale natural disasters [14]. Amy et al. proposed a dynamic influenza disease transmission model to predict the demand for antivirals for patients with pandemic influenza, providing important suggestions into the impact of the inadequate distribution of antiviral treatment [15]. The above studies mainly focus on surging demand forecasting, resource scheduling, and response measures, mostly based on deductive reasoning modeling methods or static contingency plans, lacking experimental design and experimental studies that integrate multiple uncertainties. Due to the randomness, complexity, and uncertainty of disaster evolution [16], it is difficult for the existing methods to accurately describe and predict the dynamic surging emergency product demand and effectively improve the manufacturing system.

To address the above research issues, we propose a parallel simulation and optimization framework (Section 3) based on the theories of RSS and PeMS (Section 2). This framework consists of four critical steps, including RSS-based emergency production process modeling (Section 4), parallel model cultivating (Section 5), process optimization (Section 6). Furthermore, taking the shortage of medical protective masks in the COVID-19 outbreak as an experiment background, an emergency response production process, which can supply enough masks and satisfy the surging demand effectively and timely, is obtained after modeling, cultivating, and optimization (Section 7).

## 2 Materials and methods

### 2.1 Theoretical foundation

#### 2.1.1 Parallel emergency Management System (PeMS)

Unconventional Emergency Management System can be regarded as an open and complex system. Because of its indivisibility and unknowable hypothesis [17], it is inherently difficult to predict unconventional emergencies accurately. However, the correct prediction and effective prevention of unconventional emergencies is a focus of emergency management that cannot be ignored. To solve this problem, Wang proposed the parallel computing theory for complex systems in 2004 [18]. Based on the viewpoint of multiple worlds, he proposed an artificial social model which is parallel and consistent with the real emergency system in terms of scale, behavior mode, and system characteristics. The computing experiment method is used to test the possible scenarios to evaluate the effectiveness of the artificial emergency model. By comparing and analyzing the respective future scenarios through parallel control (i.e., the actual society and the artificial society are juxtaposed to form parallel scenarios) to adjust the respective management and control, and thus the purpose of dynamic and optimal management and control is achieved. Integrating the above methods, Wang proposed an ACP methods system including Artificial Societies, Computing Experiments and Parallel Control [17], which provides a feasible way to study complex systems.

Based on the above theories, Wang proposed the theoretical framework of Parallel Emergency Management Systems (PeMS) [19,20] in 2007. The core idea of PeMS is to use the artificial society method to model unconventional emergencies, then design a computing experiment process to analyze and evaluate the emergency response measures, and finally implement the emergency plans through parallel control. Compared with the traditional "prediction-response", PeMS is suitable for the emergency management of irregular and unprecedented emergencies and has a strong "scenario-response" property, which can provide decision-making reference for scenario modeling, computing analysis, and parallel evolution optimization of unconventional emergencies.

Although the theory has contributed to emergency management research on unconventional emergencies, it also has certain limitations. The massive scale of computational experiments and parallel control based on artificial systems necessitates high-performance computing platforms and parallel computing techniques [17]. Because these conditions can lead to great modeling difficulties and computational costs, the technique is mainly used to study complex systems such as production control and traffic control.

#### 2.1.2 Random Service System Theory (RSS)

Random Service System (RSS) theory is to study how to reasonably achieve a balance between random arrival of customers and a limited number of servers [21]. Mainly through the probabilistic statistical description and related mathematical modeling of service objects’ arrival interval and service time, some quantitative indicators (such as waiting time, queue length, etc.) are derived. Then according to these indicators, the structure of the service system is improved, or the service object is reorganized so that the service system can not only meet the needs of the service object but also minimize the overall cost of the service organization or optimize certain indicators. The theory is widely used in industrial, commercial, service, and other resource-sharing random service systems. There are many queuing problems in factories, such as the arrival of orders being random and unpredictable under unconventional emergencies, while the number of processing equipment needs to be decided in advance. Too little arrangement of processing equipment will not meet the demand of order arrival, while too much arrangement faces the problem of cost [22]. Therefore, we need to scientifically and rationally set up the manufacturing systems to achieve a win-win demand and production services situation.

Inspired by this research idea, the surging emergency demand for medical protective masks and the manufacturing capacity of a factory under unconventional conditions are considered as a queuing service system in this paper. The theory of RSS is applied to model the production processes. Then the model can be analyzed and cultivated in a parallel way to find the effective manufacturing system structure for satisfying the surging emergency production demand.

### 2.2 Parallel simulation and optimization framework of emergency production process based on RSS-PeMS

According to the ACP theory, the basis of parallel emergency management is to construct the artificial society of dynamic scenarios [17]. Real-time data from real scenarios are used to evolve artificial social models so that various emergency decision-making plans can be extrapolated based on the latest trends, and changes in events can be timely predicted. Plans are then dynamically adapted and developed to assist decision-makers in proposing response measures and implementing appropriate actions [23].

**Fig 1:**
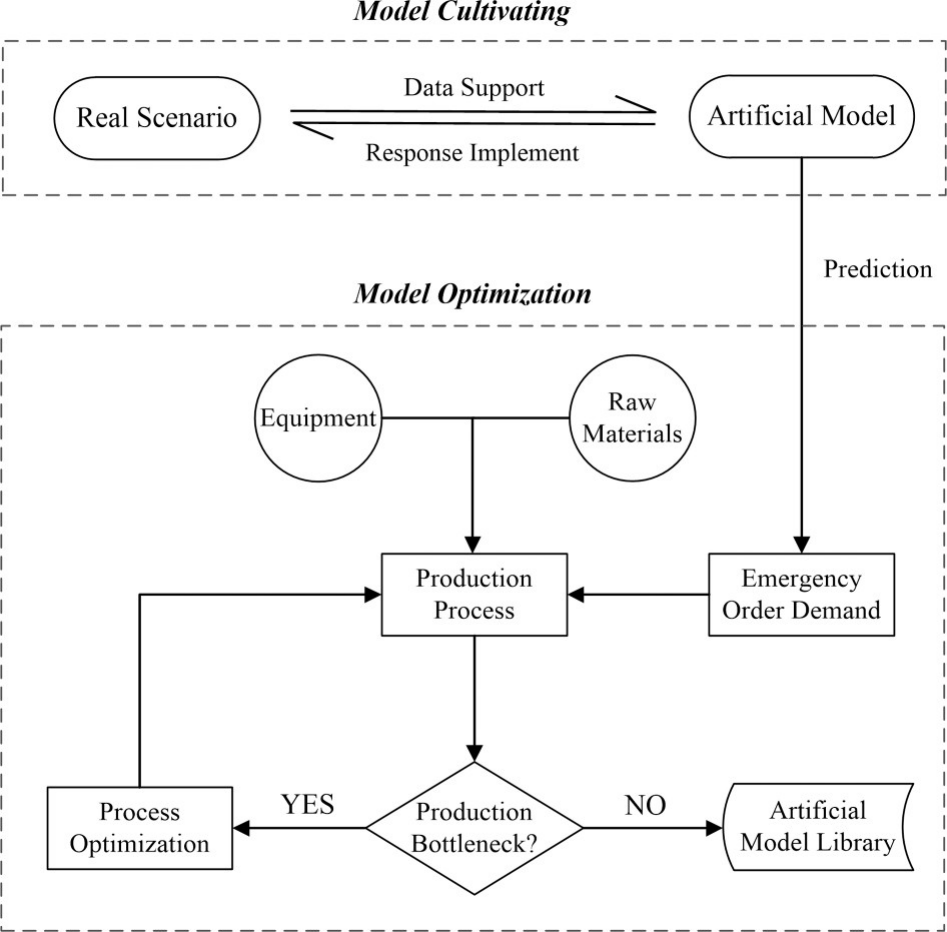
Parallel simulation and optimization framework based on RSS-PeMS.

According to the emergency production process of factories in the realistic epidemic scenario [24], the following scenario elements should be constructed in the artificial society: ①Emergency Order Demand (EOD); ②Production Equipment (PE); ③Production of Raw Materials (PM); ④Production Process (PP). These interacting scenario elements form the RSS-PeMS simulation and optimization framework, as shown in Fig 1. Firstly, the emergency material demand data of each stage in the real epidemic scenario are obtained, collated, and analyzed, and the modeling is conducted using suitable technical methods (e.g., Python) and simulation tools (e.g., FlexSim). Then the parallel implement process is used to obtain a model that can effectively simulate the real scenario, and this model predicts the EOD of the next stage. With the support of PE and PM, the production process system is run according to EOD. Determine whether there is a bottleneck process in the current production process and optimize it. Finally, the optimized production process is applied to the production process of the next stage of the epidemic. It is iterated and optimized according to the real-time data of the real scenario until it can effectively respond to the surge in EOD, providing the artificial model library with production processes that can be used for the next unconventional event.

In addition, several assumptions required in the parallel simulation and optimization framework are added as follows: Firstly, the data of the Real Scenario must be real and available in time to ensure the accuracy and credibility of the future Real Scenario EOD deduced from the Artificial Model supported by the Real Scenario real-time data. Secondly, the Production of Raw Materials needed in the production process is sufficient, and the production equipment and workers’ processing efficiency is stable so that the model can run properly. Finally, given that the cost issues involved during the optimization of the production process are not the focus of this paper, they are not considered in this paper for the time being.

### 2.3 RSS-based emergency production process modeling (Section 4)

#### 2.3.1 Definition of simulation entities and relationship flow

**Fig 2:**
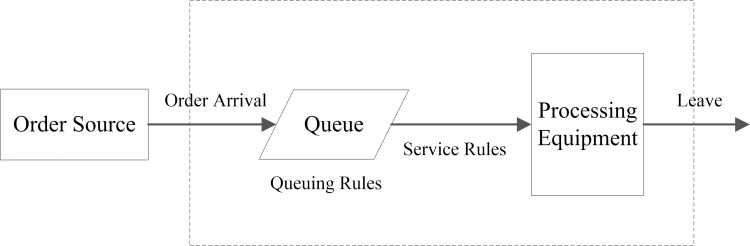
General simple manufacturing process model [25].

According to the RSS theory, a general simple manufacturing process model is shown in Fig 2 [25]. Firstly, the order from the order source to the manufacturing system is a random input process. And then, the order is transformed into the products to be processed and waited for processing based on the queuing and service rules. Finally, as an output process, the products leave the queuing system after processing services.

Based on the RSS theory, we construct the simulation model from the perspectives of Fixed Entity (FE), Temporary Entity (TE), and Relationship Flow (RF) according to the general simple manufacturing process model.

FE represents the main executors in the emergency manufacturing process, and such entities do not readily change as scenarios evolve. For example, PE performs production tasks at a stable service rate in the simulation system, a Fixed Entity (FE_PE_). TE represents the object to be processed, and its state often changes with the evolution of the situation. For example, EOD and PM, which change with the change of emergency production process at the arrival rate and processing rate of each interval, are defined as TE_EOD_ and TE_PM_.

After determining the entity elements of the simulation model, we establish the Relationship Flow between the elements with reference to the emergency production process. Based on the three basic components of the queuing system (input process, queuing rules, and service institutions), we classify the Relationship Flow into Input Flow (IF), Waiting Flow (WF), and Service Flow (SF).

The serviced objects arrive at the simulation system individually or simultaneously according to certain arrival rules, defined as the IF. In this paper, EOD (which can be denoted as IF_EOD_ in terms of Input Flow) arrive successively at the simulated production system at independent time intervals and obey the negative exponential distribution. Their arrival probabilities can be expressed by the formula: *P* = 1 – *e*^-*λt*^ (λ represents the arrival rate of the orders). After entering the production system, orders are converted into products to be processed and waited for processing according to certain queuing rules. This process is defined as a WF. In this paper, for the products to be processed (i.e., raw materials to be processed, denoted as WF_PM_), the waiting queuing rule of First-Come First-Served (FCFS) is adopted. To be processed products obey certain service rules and are transformed into semi-finished or finished products through the service organization’s processing, defined as SF. According to the queuing network theory [26], the simulation model in this paper is a series-parallel queuing network system. The processing time of each Production Equipment (SF_FE_) at each node obeys the normal distribution, and the formula is expressed as: $f(t)=\frac{1}{\sqrt{2\pi }\sigma }e^{-\frac{{\left(t-\mu \right)}^{2})}{\left(2\sigma^{2}\right)}}$ (μ represents the processing rate of PE, and σ represents the standard deviation of the processing time). On the basis of the above, we can use IF_EOD-PP_ to represent the emergency demand orders entering the simulation production process, and the arrival rate is: $\lambda =\frac{D}{t}$ (D refers to the number of orders entering the production process in time t). WF_PM (m)-PE(m)_ means that the mth production raw material is about to flow into the mth production equipment to be processed. The average queue length and average waiting time are obtained as follows: ${\text{L}}_{\text{q}}=\frac{\frac{\lambda^{2}}{c^{2}{}_{i}\mu^{2}}+\lambda^{2}\sigma^{2}}{2*\left(1-\frac{\lambda }{c_{i}\mu }\right)}$; $W_{q}=\frac{L_{q}}{\lambda }$ (c_i_ represents the number of devices for the ith procedure). SF_PM (m) -PE (m)_ means that the mth production equipment is processing the mth production raw materials, and the service intensity of the production equipment is: $\rho =\frac{\lambda }{c_{i}\mu }$. Moreover, the input quantity of emergency demand orders should not be greater than its output quantity, and the input quantity of production raw materials needs to be not less than its output quantity.

#### 2.3.2 Emergency production process modeling

**Fig 3:**
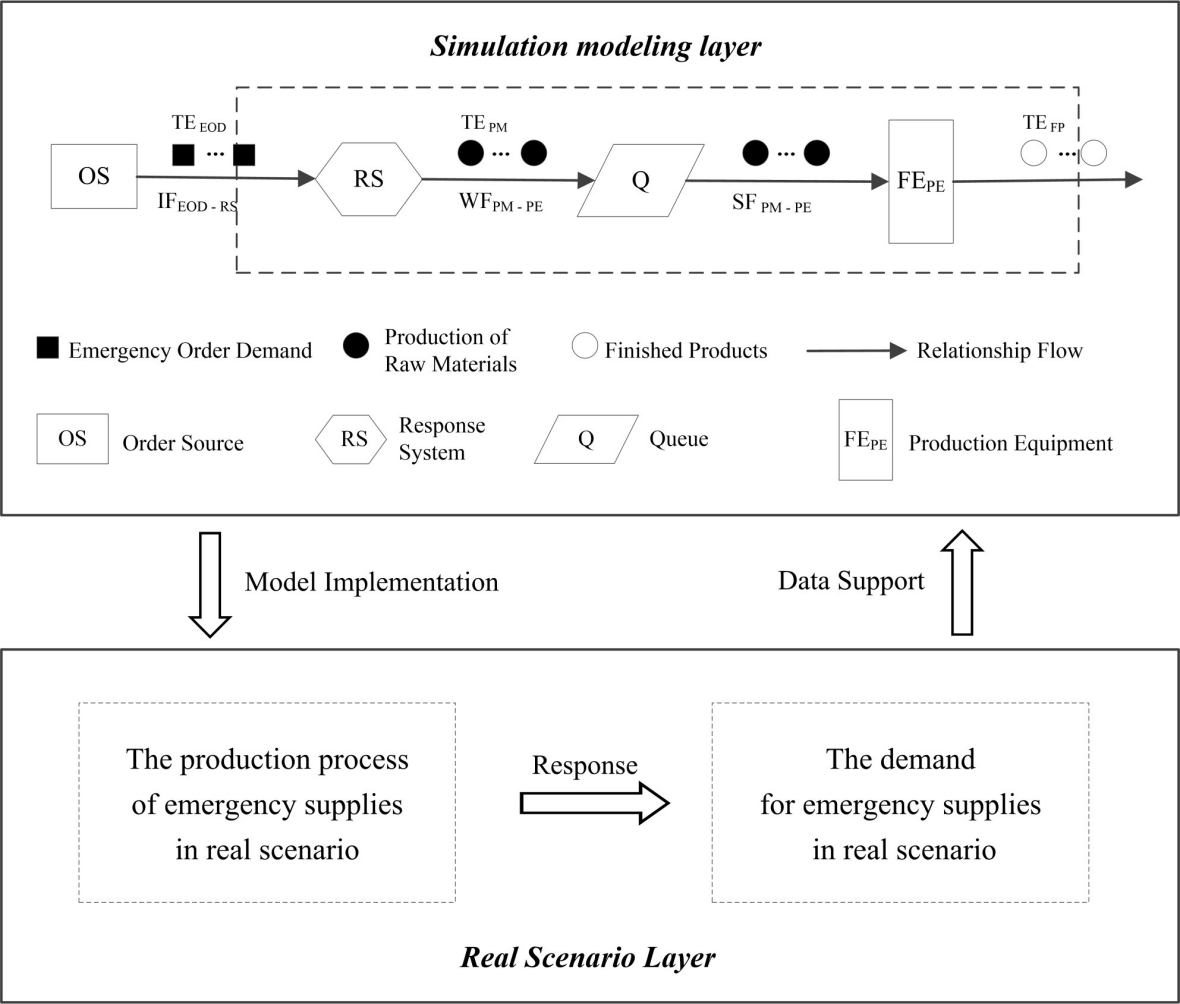
Emergency production process simulation model.

According to RSS and Queuing Network Theory, the emergency production process simulation model is established, including two levels of real scenario layer and simulation modeling layer, as shown in Fig 3. In the real scenario layer, this paper reflects the demand for emergency supplies in a realistic epidemic scenario, and the supplies and production processes used for emergency response. The real scenario is not only the data source of the parallel simulation modeling layer, but also the target of simulation process optimization. In the simulation modeling layer, we layout the series-parallel queuing network system of the simulation production process based on the data provided by the real scenario [23] (including the entities of the process and their relationship flow), as shown in Fig 3. The FlexSim simulation tool is used to simulate the real scenario to build the model, and the implementation method is shown in Table 1. Firstly, for fixed entities (such as production equipment) and temporary entities (such as production raw materials), we can use Fixed Resources (such as Processor, etc.) and Flow Items (such as Box, etc.) in the FlexSim tool to implement the relevant functions, respectively. Then, the model and performance of simulation entities in the production process system are simulated by setting and adjusting relevant parameters. For the three relationship flows (IF, WF, SF) between entities, the "Port Connection" function can be implemented. Finally, the simulation model is loaded and run. Thus, an RSS-based emergency production process model for simulating the surging orders can be constructed through the above steps.

**Table 1:** Real scenario elements and simulation model elements corresponding to FlexSim.

Simulation Model Elements	Real Scenario Elements	Related Elements in FlexSim
Fixed Entity (FE)	Production Equipment	Fixed Resources: Processor, Combiner, Separator, etc.
Temporary Entity (TE)	Emergency Order Demand; Production of Raw Materials	Flow Items: Box, etc.
Relationship Flow (RF)		Port Connection

### 2.4 Parallel model cultivating (Section 5)

**Fig 4:**
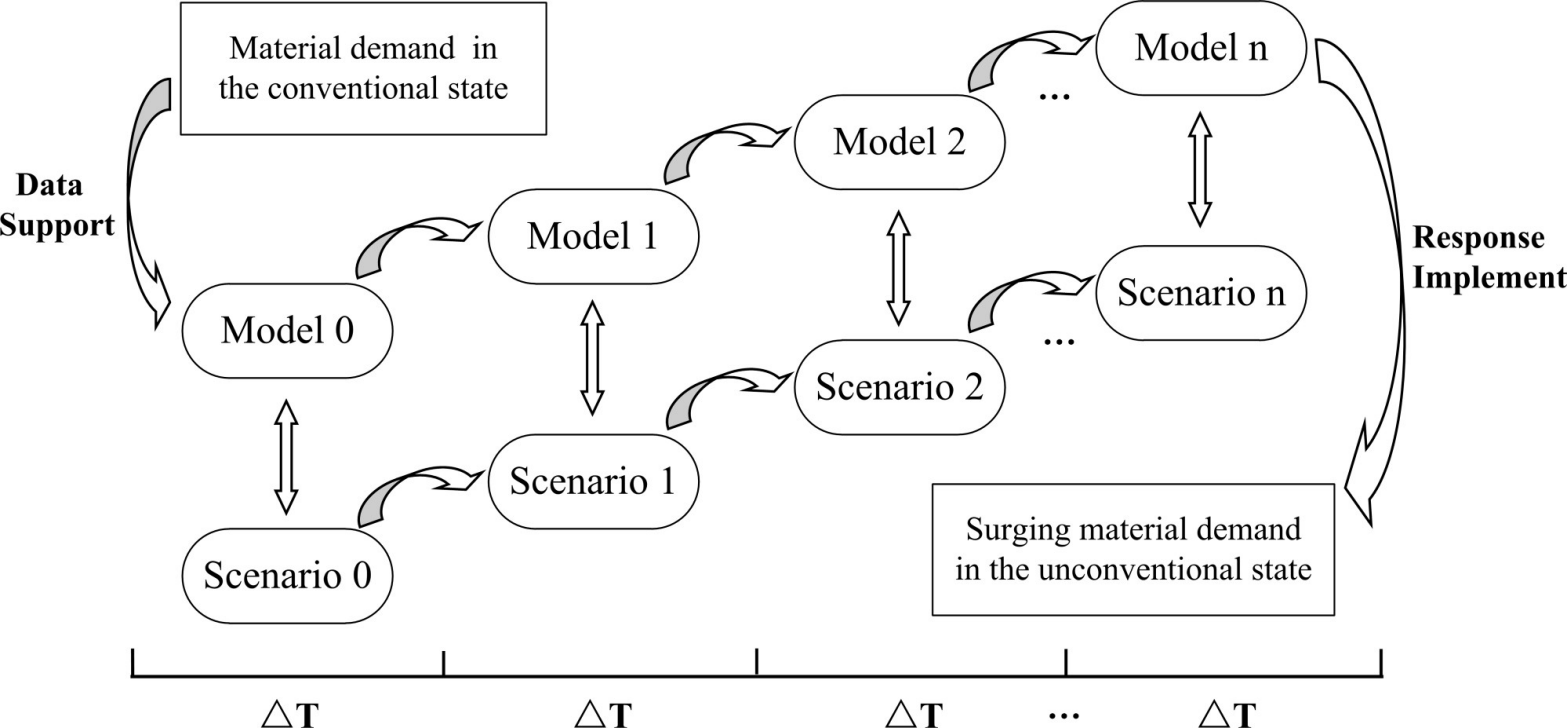
Parallel implementation mechanism for surging demand.

Based on the emergency production process simulation model, a parallel system containing artificial scenarios and real scenarios can be established to realize the parallel implementation of emergency response requirements based on RSS-PeMS. Its parallel implementation mechanism is shown in Fig 4. Firstly, the artificial scenario in the conventional state and the real scenario in the unconventional surge state are set as simulation model *Scenario* and *Model*, respectively. The total simulation time is T_n_ (ΔT is the simulation time interval of each stage and T_n_ = n ΔT). Then, through the real-time collection, collation, and data analysis, the artificial scenario simulation model *Model 0* in the conventional state and the real scenario simulation model *Scenario 0* in the Surging demand state are established. Based on each simulation time interval ΔT, the differences between artificial scenarios (*Model 0 ~ Model n*) and real scenarios (*Scenario 0 ~ Scenario n*) are compared and analyzed. Regression analysis is used to fit the model parameters to modify the artificial scenario simulation model gradually (i.e., train a linear regression model based on historical data of order arrival intervals of the real scenario, and then use the model to predict the parameter values for the next order arrival interval of the artificial scenario until the artificial model can simulate the real scenario). To evaluate artificial model’s authenticity, the predicted values are compared with the measured values based on the Goodness-of-Fit test method [27]. The calculation formula is as follows:

$$R^{2}\left(y,\hat{y}\right)=1-\frac{\sum {\left(y_{i}-{\hat{y}}_{i}\right)}^{2}}{\sum {\left(y_{i}-{\bar{y}}_{i}\right)}^{2}}$$

If the value of *R*^*2*^ is more than 85% (i.e., GOF > 85%) [28], it can indicate the validity of the predicted value. Moreover, the closer *R*^*2*^ is to 1, the closer the relationship between the measured value and the simulated value is, and the better the fit is. It also means that the cultivated artificial model is very close to the real scenario. The decision-makers can use it to simulate and predict the future change of emergency order demand and make accurate and effective emergency decisions.

### 2.5 Process optimization (Section 6)

The surging emergency orders cause the production lines to be overloaded or even paralyzed. According to the RSS theory, we can identify a process as the bottleneck process of a production line if the average number of products waiting for a process (AvgWIP) is too high or the average waiting time for products to be processed (AvgStaytime) is too long. The decision-maker can make the process more efficient by reducing the AvgWIP or AvgStaytime. At present, there are two approaches to make the whole production process smoother. On the one hand, we can directly observe the production process under the surge in demand with the help of the cultivated simulation model (shown in Section 5). Based on the simulation results, the rationality of the production line in terms of spatial position and equipment number is observed and analyzed. The corresponding optimization plan can be formulated after discovering the processing link of severe congestion, which is the bottleneck link affecting production efficiency. On the other hand, after deriving the result data from the simulation model, the bottlenecks that affect productivity can be quantitatively analyzed, and the allocation optimization can be realized effectively. There are two main optimization methods at least. One is to increase the production equipment of the production line (change the process structure), which needs to consider the cost of additional equipment and how much benefit it can bring to the factory. The other is to increase the service rate of production equipment or workers. The optimization methods should be based on analyzing the factory’s own situation and striving to achieve the global optimal production line.

## 3 Results (Section 7)

### 3.1 Example background

In early December 2019, a sudden outbreak of the COVID-19 pandemic occurred in Wuhan, Hubei Province. The pandemic spreads rapidly across the country in a short time with its extremely infectiousness, which led to a severe shortage of emergency supplies in Wuhan and even the whole country. We take the surging demand for medical protective masks during the COVID-19 as the example background and build a simulation model to interpret the changes of the scenario in real-time and find out its dynamic evolution rules to optimize the manufacturing process. All the data are collected from the official website of the National Health Commission of the People’s Republic of China [29].

### 3.2 Modeling and simulation

By processing the data published on the official website of the National Health Commission of the People’s Republic of China, we can conclude that 10.08 million [29] is the demand for medical protective masks in the Huoshenshan Hospital for one week(The usual stock of masks in the hospital is about one week’s supply)[30]. Since the order arrival meets the characteristics of stability, non-inferiority, and rarity, the negative exponential distribution can be used to simulate the order arrival pattern. Combined with the above data, the initial mean value of the order arrival time interval can be temporarily set to 17.14 minutes in the initial model. The order arrival time interval parameters will be improved by the parallel iterative process of the subsequent model. By Extracting and processing the data of the mask production line released by Xintaiming Intelligent Equipment Co., the processing process and processing schedule are derived as shown in Table 2 [24].

**Table 2:** Processing time of each process.

Process	Processing Time
Laminating, Knurling, Cutting and Shaping	Normal (0.75, 0.16,0) s
Sealing	Normal (2.40,0.04,0) s
Welding Ear Bands	Normal (3.43, 0.02,0) s
Welding Brackets of Nasal Bridge	Normal (4.00, 0.04,0) s
Packing	Normal (1.00,0.00,0) s
Sterilization and Resolution of Ethylene Oxide	22 hours per batch

The *Mean* and *Standard Deviation* in Normal (*Mean*, *Standard Deviation*, *Stream*) refer to the mean and standard deviation of the seconds used to process a product in a certain process. According to the set requirements of the software, *Stream* did not play a role in this experiment, so the default setting is 0.

**Fig 5:**
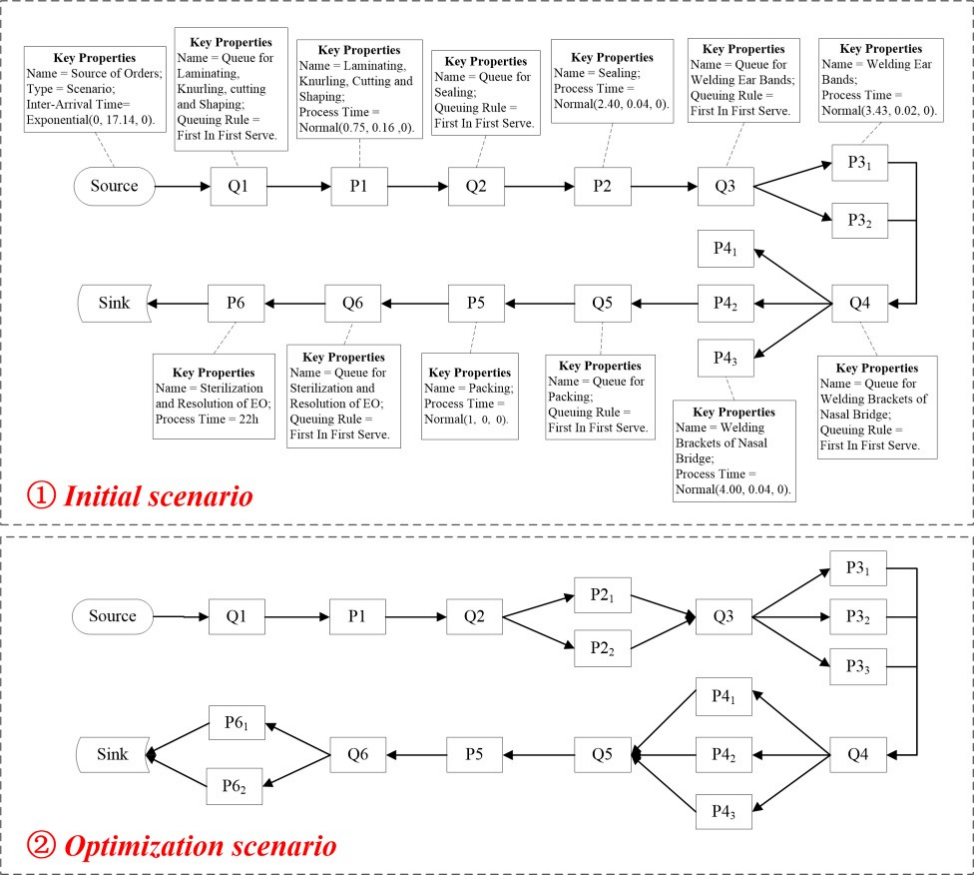
Production line scenario simulation model.

**Fig 6:**
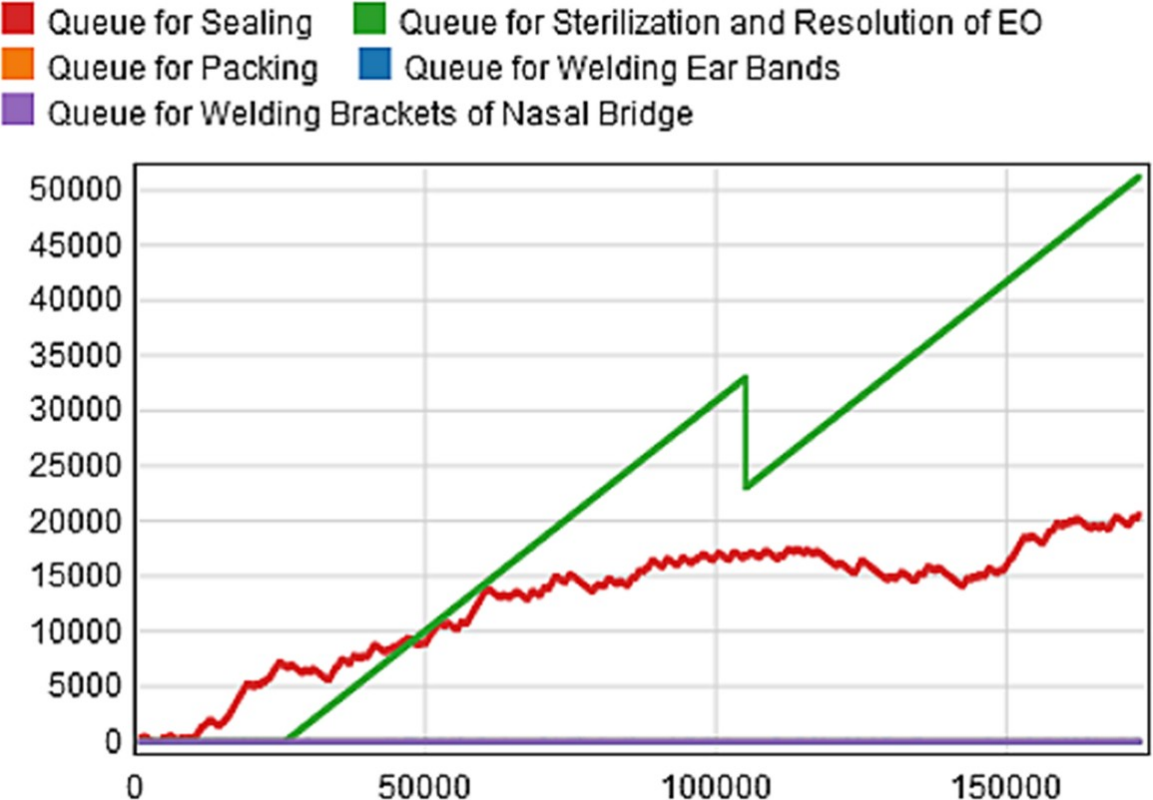
Scenario staging area "Time-Capacity".

Based on these parameters, we construct the initial scenario of the production line model as shown in Fig 5-①. Given that the interval between the press conference announcing the official completion of the hospital and its commissioning is 48 hours, we temporarily set the response time to 48 hours [29], and then use the simulation tool FlexSim2020 to run the model for 48 hours (i.e.2880 minutes). The Queuing information of each process is counted, and a relevant "Time-Capacity" diagram is drawn, as shown in Fig 6.

### 3.3 Parallel cultivation

This experiment assumes that the production efficiency of the equipment and the work efficiency of the operators used for the production of medical protective masks are stable, so we set the distribution function obeyed by the processing time of each production link to be constant. In addition, if the number of products to be processed in the staging area is used as a criterion, we can easily find that the number of products to be processed in the staging area before the two processes of "Sterilization and Resolution of Ethylene Oxide" and "Sealing" is the largest according to Fig 6. Therefore, we selected "Queue for Sterilization and Resolution of EO" and "Queue for Sealing", which are two staging areas with congestion, as the main objects of our study. The total simulation time of this experiment is 2880 minutes. By taking "ΔT = 480 minutes" as a stage length, we can cultivate six generations of models (*Model 0* ~ *Model 5*). Then, the simulation results of parallel models with real scenarios are gradually compared in each stage. Finally, the model is gradually adjusted and improved by fitting parameters to make it real and reliable.

#### (1) *Model 0* modeling and simulation

**Fig 7:**
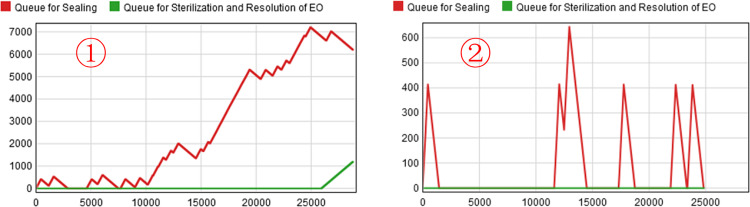
*Scenario 0* (left), *Model 0* (right) Staging Area "Time-Capacity".

**Fig 8:**
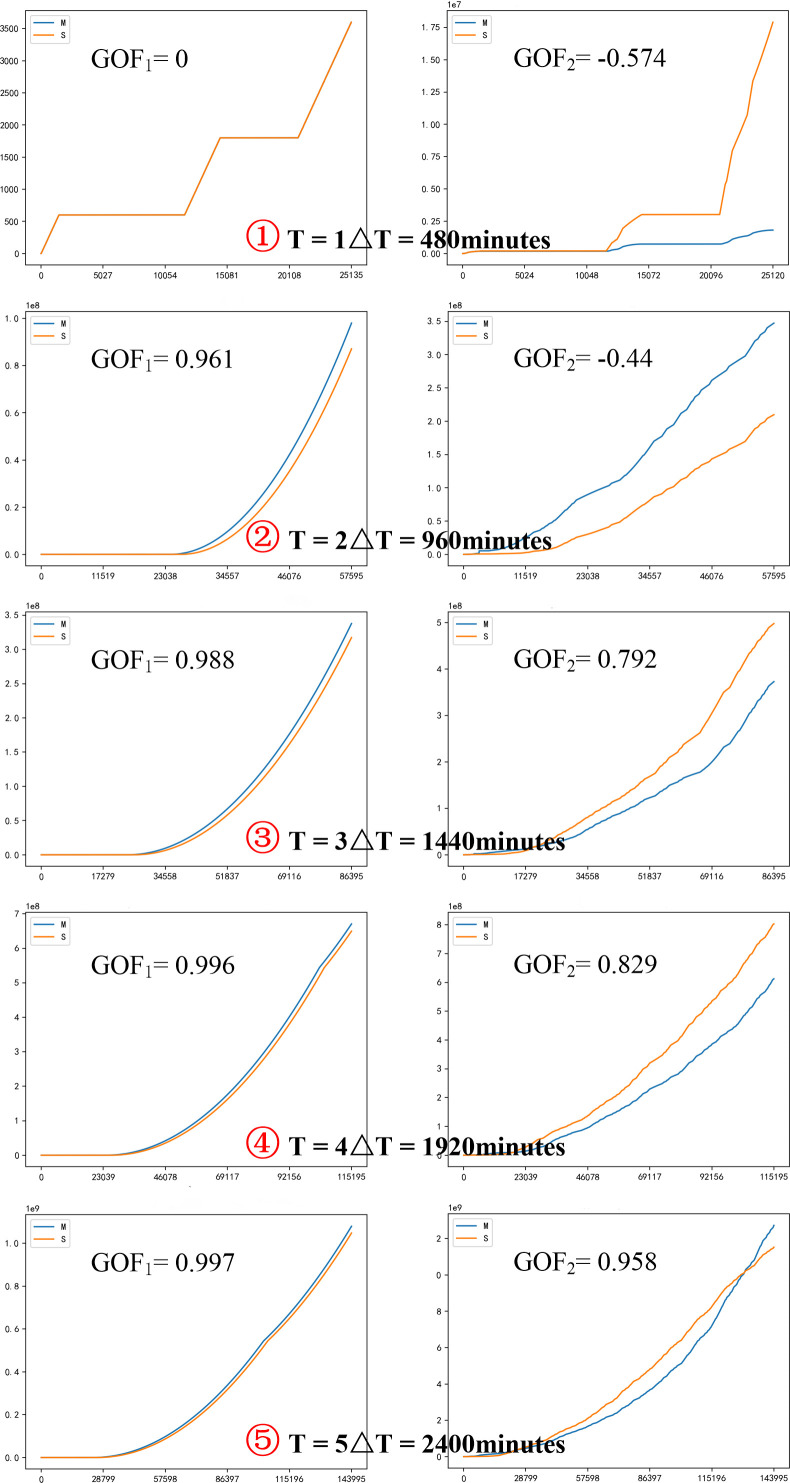
*Model 0*~*Model 4* demand trend evolution.

By processing the collected data, we know that the order arrival time interval of medical protective masks in the conventional state obeys the negative exponential distribution with a mean value of 172.46 minutes, i.e., the order arrival interval of the first-generation model (*Model 0*) obeys Exponential (0,172.46,0). The model is run for 480 minutes (1ΔT) to plot the "Time-Capacity" diagram of the staging area as shown in Fig 7-② and the evolution of the demand trend as shown in Fig 8-①. By observing Fig 7-②, we can see that the maximum waiting queue value for the products to be processed does not exceed 650, and there is no congestion in the staging area. Therefore, we can assume that the model can respond effectively to the epidemic. However, by observing the congestion of Fig 7-① *Scenario 0* in the same period (0 ≤ t < 480 minutes), we can find that the congestion of the real scenario *Scenario 0* is much more severe than that of the artificial scenario *Model 0*. To compare the differences between the two scenarios more accurately, we processed the model data in Python and programmed based on the "Goodness-of-Fit formula" to test the reality of the model. Using "GOF_1_" and "GOF_2_" to represent the GOF values of "Sterilization and Resolution of Ethylene Oxide" and "Sealing", respectively, we calculate that GOF_1_ = 0 and GOF_2_ = -0.574, both fail to meet the criterion of GOF>85% [28], which indicates that the first-generation simulation model (*Model 0*) does not representative of the real scenario *Scenario 0*, so it still needs to be further adjusted and optimized.

#### (2) *Model 1* modeling and simulation

According to the sudden and surge characteristics of the COVID-19, we judge that the order average arrival time interval of *Model 0* is over large, which leads to inconsistent simulation results with *Scenario 0*. To ensure consistent simulation results, we use regression analysis to predict the mean value of the order arrival time interval in the next stage. A sequence of simulated values with different coefficients are obtained, and then the one with the minimum error is selected as the mean value of the order arrival time interval. After analysis and calculation, we find that the simulated value sequence with the mean value of 14.54 has the smallest error, so the order arrival time interval distribution function of the first-generation model (*Model 0*) is adjusted to Exponential (0,14.54,0), and the second-generation model (*Model 1*) is obtained. Then the new model is run for 960 minutes (2ΔT), and the trend evolution of demand is plotted as shown in Fig 8-②. Similarly, using the above methods to calculate GOF values, we obtain GOF_1_ = 0.961 and GOF_2_ = -0.44. Although the GOF values are improved compared with the previous generation model, but don’t meet the requirement of GOF>85% [28]. Thus, they still cannot simulate the real scenario (*Scenario 1*) and thus cannot accurately predict the evolution trend of the demand for medical protective masks.

#### (3) *Model 2 to Model 4* modeling and simulation

Similarly, we perform the same operation on *Model 2* to *Model 4*, improving the model from the third-generation (*Model 2*) to the fifth-generation (*Model 4*), and obtain the following data: the order arrival time intervals of *Model 2*~*Model 4* obey Exponential (0,16.41,0), Exponential (0,16.57,0) and Exponential (0,17.13,0), respectively. Then the models were run for 1440 minutes (3ΔT), 1920 minutes (4ΔT), and 2400 minutes (5ΔT). The evolutions of demand trends are plotted as shown in Fig 8-③~8-⑥. Finally, the GOF values of each stage are calculated as follows: GOF_1_ = 0.988 and GOF_2_ = 0.792, GOF_1_ = 0.996 and GOF_2_ = 0.829, GOF_1_ = 0.997 and GOF_2_ = 0.958, respectively. It is evident that the values of GOF_1_ and GOF_2_ increase during the iterations, and the GOF values of the cultivated fifth-generation model (*Model 4*) have exceeded the standard of GOF>85% [28]. Therefore, we believe that the cultivated *Model 4* can better simulate the real scenario and can be used to predict the future demand for medical protective masks and simulate the manufacturing system performance.

### 3.4 Process optimization

**Fig 9:**
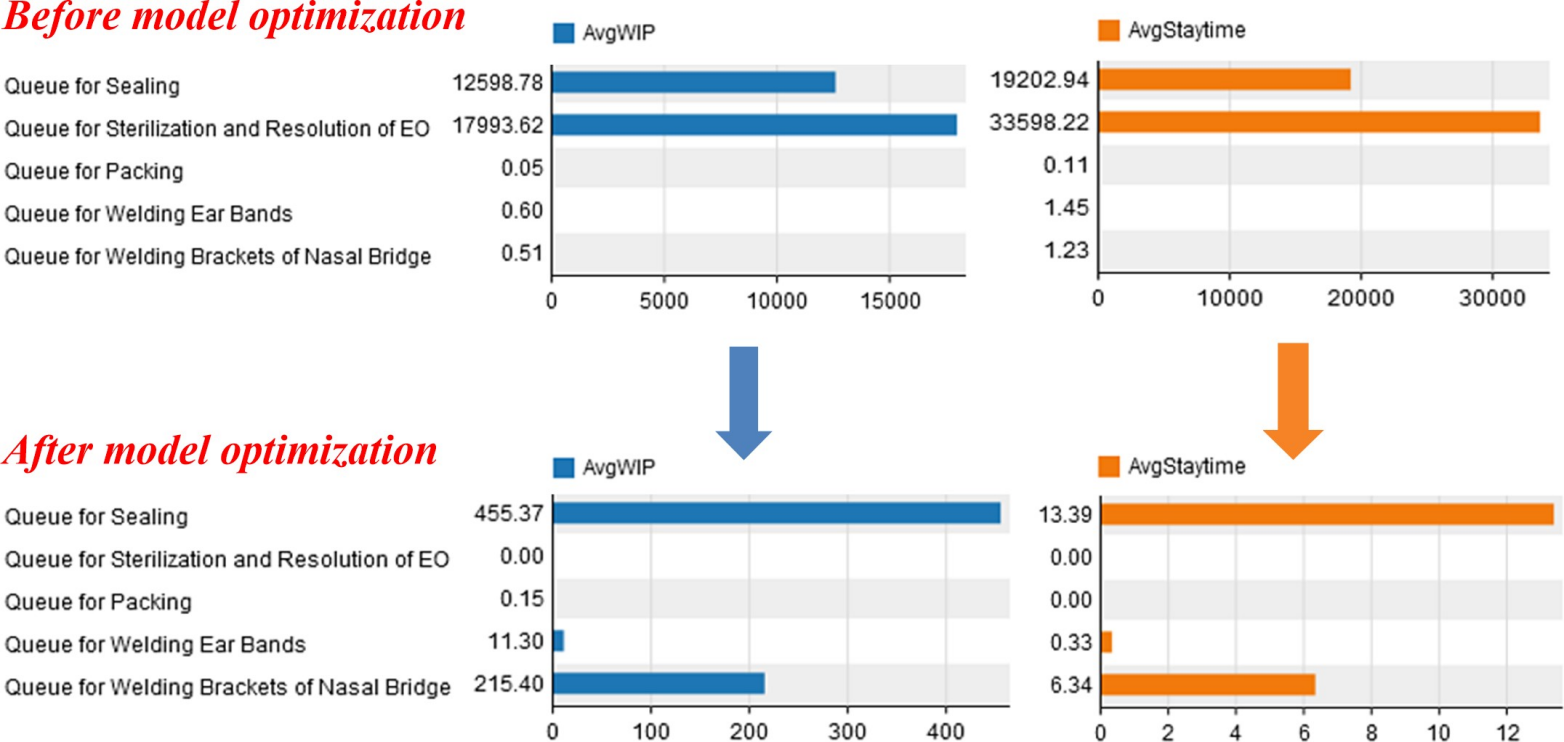
Comparison of AvgWIP and AvgStaytime before and after *Model 4* optimization.

In order to make the production process work normally, decision-makers can improve the bottleneck process based on the principles and methods described in Section 6. Counting the AvgWIP and AvgStaytime of the staging area before each link of the production process in the *Model 4*, the statistical diagram of Fig 9 is obtained. It is not difficult to find that "Queue for Sterilization and Resolution of EO" and "Queue for Sealing" are the two links with the most AvgWIP and the longest AvgStaytime. It can be seen from Fig 5-① that there are two reasons for the congestion: firstly, there is only one piece of equipment for the procedure of "Sealing" and the processing time is long; secondly, the procedure of "Sterilization and Resolution of Ethylene Oxide" adopts a small (2 m^3^) Ethylene Oxide sterilizer, which can only process 10,000 masks at a time and takes 22 hours to sterilize and resolve once. The example focuses on the optimization of the above two problems. According to the process optimization methods described in Section 6, we optimize the model on the basis of *Model 4* to obtain *Model 5*. The specific optimization methods are as follows: first, one mask sealing machine and one ear band welding machine are added in the two processes of "Sealing" and "Welding Ear Bands", respectively. Then, the small (2 m^3^) Ethylene Oxide sterilizer used in the "Sterilization and Resolution of Ethylene Oxide" process is upgraded to two medium (6 m^3^) Ethylene Oxide sterilizers. Finally, the process line optimization scenario is obtained as shown in Fig 5-②. Moreover, by counting the data of AvgWIP and AvgStaytime in each staging area after the optimization process, the statistical chart is drawn as shown in Fig 9. According to Fig 9, it is obvious that the optimized AvgWIP and AvgStaytime are significantly reduced compared with those before optimization. Therefore, we believe that the congestion on the production line has been effectively relieved compared to the *Model 4* without optimization.

**Fig 10:**
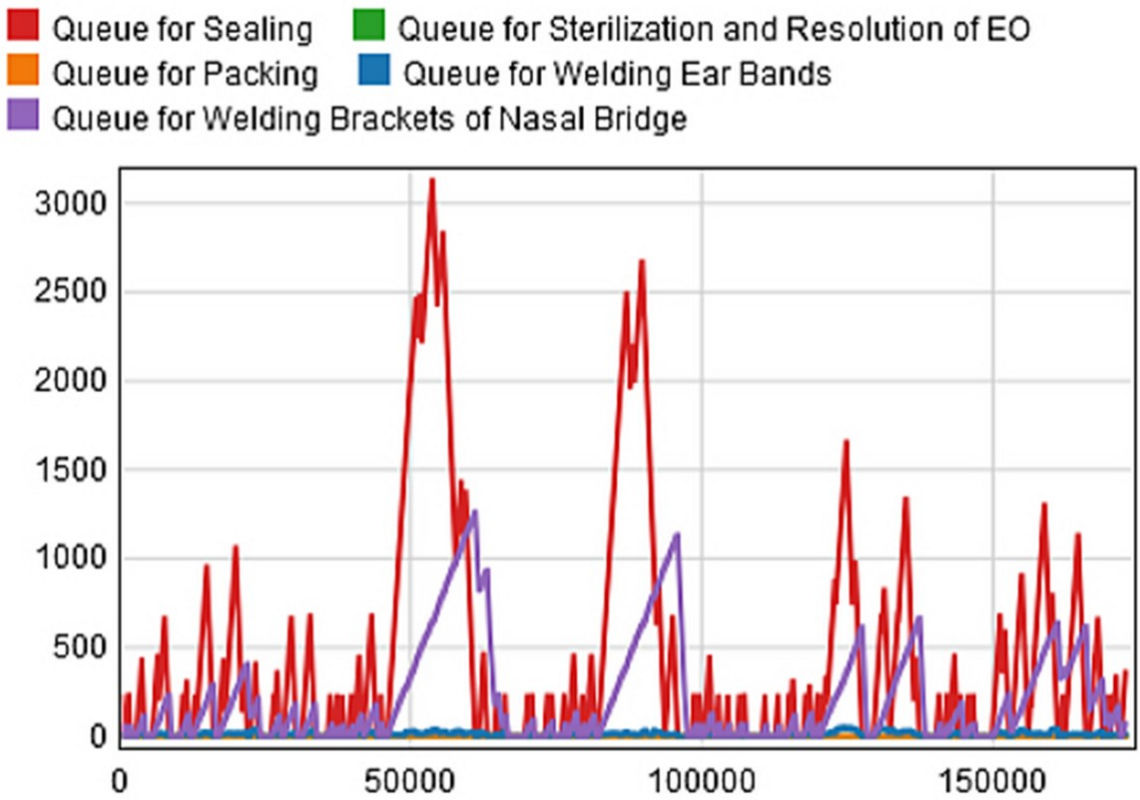
*Model 5* staging area "Time-Capacity".

To further evaluate the optimization effect, we compare the optimized model with the real scenario, so run the optimized model for 2880 minutes (6ΔT) and plot the "Time-Capacity" of the staging area as shown in Fig 10. Observing and comparing the congestion of *Scenario 5* (Fig 6)in the same period (0 ≤ t < 2880 minutes), we can see that the peak value of *Model 5* is significantly smaller and the evolution trend is gradually slower, indicating that the model can effectively solve the problem of surging demand under the epidemic.

The optimization method of increasing production equipment mentioned above belongs to the first way, and we can also adopt the second way. However, both have their own advantages and disadvantages. The former may be limited by production costs, while the latter may lead to physical strength overdraft of workers. Thus, when deciding which specific optimization way to use, the real situation of a factory should be taken into account to achieve the best production state.

## 4 Discussion

### 4.1 Theoretical discussions

As mentioned in the "Introduction" section, most scholars’ research on the surging demand is mainly based on deductive reasoning modeling methods or static contingency plans for demand forecasting and resource scheduling, which are less able to obtain sufficient model parameters to build and dynamically adjust the model in a sudden, complex, dynamic and unconventional outbreak scenario like the COVID-19 epidemic. Compared with the modeling approaches developed by these scholars, the parallel simulation and optimization framework proposed in this paper based on RSS-PeMS is more advantageous in dynamically evolving disaster scenarios. Use the idea of parallelism between real scenarios and artificial models—building and iteratively optimizing artificial models based on real-time data [17], which can better simulate the actual needs of real scenarios, solving the problem that the previous methods are difficult to dynamically model and analyze the dynamic demand that evolves with disasters in complex and changing epidemic scenarios. Therefore, this paper provides new ideas for scholars in this field to study the surging demand under unconventional emergencies.

### 4.2 Limitations and future studies

Although the method proposed in this paper can effectively solve the surging demand, there are still some limitations. Firstly, the framework cannot be implemented without a premise: Parallel emergency production process optimization is carried out under a sufficient supply of raw materials for the product. However, there is a gap between this condition and the actual supply of raw materials for masks. The following research direction is how to optimize the production process in the case of raw material supply changes. Secondly, in the optimization scheme of the product production process, this paper adjusts the equipment configuration based on RSS theory, but for the sake of concise empirical study, this strategy does not take into account the cost issues caused by upgrading equipment or adding equipment. Therefore, in the subsequent studies, cost control can be added to make the model closer to reality. Finally, compared with other previous epidemics, the COVID-19 epidemic has a longer duration, a more complex evolutionary trend, and a wide range of factors affecting changes in material requirements. For example, a sudden mutation of the virus in the epidemic could cause a surge in demand for supplies that had been changing more regularly, or an effective government control action could cause the demand to drop sharply. The research data in this paper comes from the early stage of the epidemic, which means that the scenario inference of the model by the initial data alone is not enough, and its prediction of the real scenario may produce some errors. Thus, it is necessary to update data in real-time and expand the scope of data collection to improve the accuracy and reliability of the model.

### 4.3 Conclusion

The problem of surging demand for supplies caused by unconventional emergencies is an important research content of emergency management. To address the essential difficulty that the traditional "Prediction-Response" decision method is challenging to conduct in experimental studies of real scenarios, this paper establishes a parallel emergency simulation framework based on RSS-PeMS for real and artificial scenarios under surging demand. Through parallel implementation and model cultivating, the artificial model can continuously approximate the real scenario as the simulation duration advances. The object-oriented visual modeling method is then used to realize the rapid modeling and dynamic optimization of emergency response under the scenario of surging demand. The emergency plan is timely obtained to respond to the imbalance between supply and demand of emergency supplies, to solve the problem of insufficient protective supplies for the staff working on the front line and for the people struggling to fight the epidemic, and to minimize casualties and economic losses due to lack of emergency supplies in the COVID-19 or possible future epidemics.

## Supporting information

S1 Fig Parallel simulation and optimization framework based on RSS-PeMS. (PDF)

S2 Fig General simple manufacturing process model. (PDF)

S3 Fig Emergency production process simulation model. (PDF)

S4 Fig Parallel implementation mechanism for surging demand. (PDF)

S5 Fig Production line scenario simulation model. (PDF)

S6 Fig Scenario staging area “Time-Capacity”. (PDF)

S7 Fig *Scenario 0* (left), *Model 0* (right) Staging Area “Time-Capacity”.
(PDF)

S8 Fig *Model 0*~*Model 4* demand trend evolution. (PDF)

S9 Fig Comparison of AvgWIP and AvgStaytime before and after Model 4 optimization. (PDF)

S10 Fig *Model 5* staging area “Time-Capacity”. (PDF)

S1 Table Real scenario elements and simulation model elements corresponding to FlexSim. (PDF)

S2 Table Processing time of each process. (PDF)
