# Supplementary figures and images for: Parallel simulation and optimization framework of supplies production processes for unconventional emergencies
Source: PLoS One. 2022 Jan 13;17(1):e0261771. doi: 10.1371/journal.pone.0261771 (PMC8758009; doi:10.1371/journal.pone.0261771)

### *Model Cultivating*

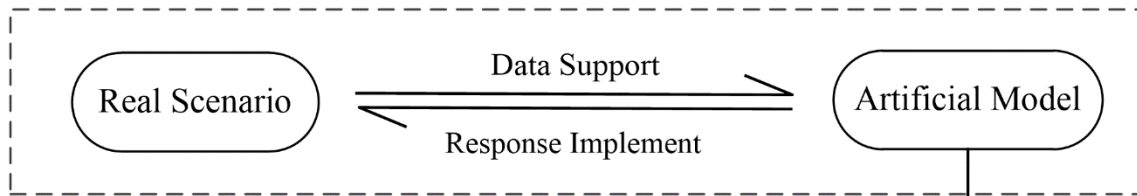

### *Model Optimization*

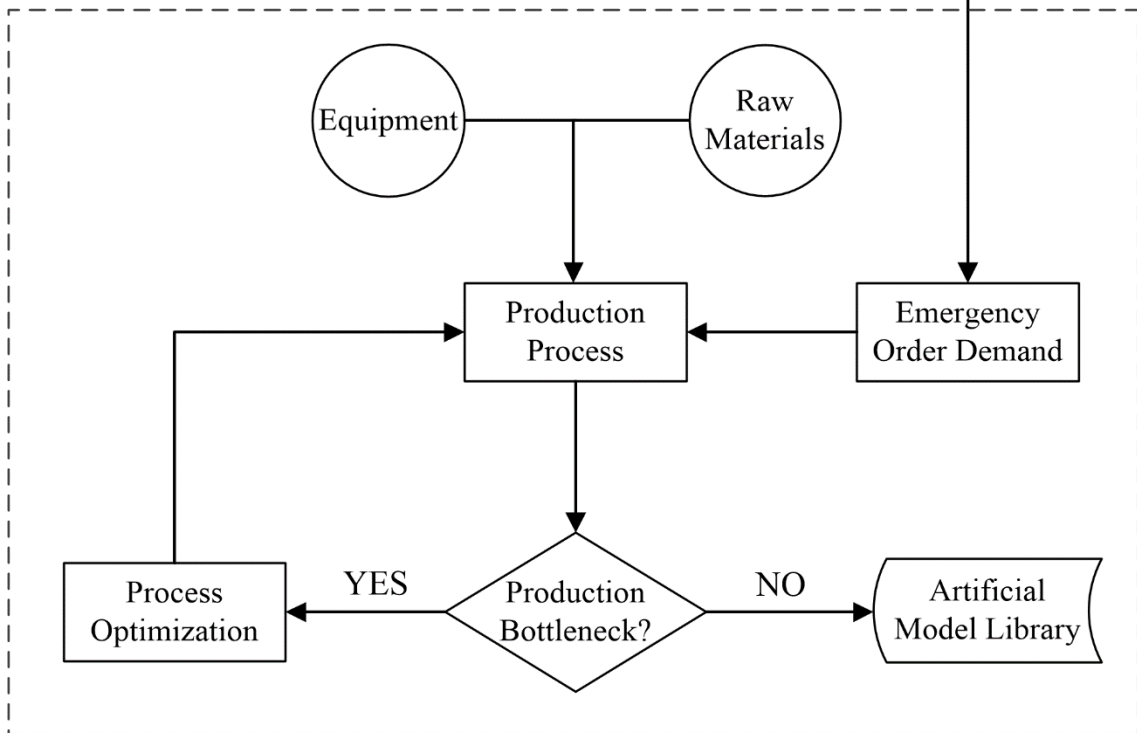

Supplement: S1 Fig — (PDF) [file pone.0261771.s001.pdf]

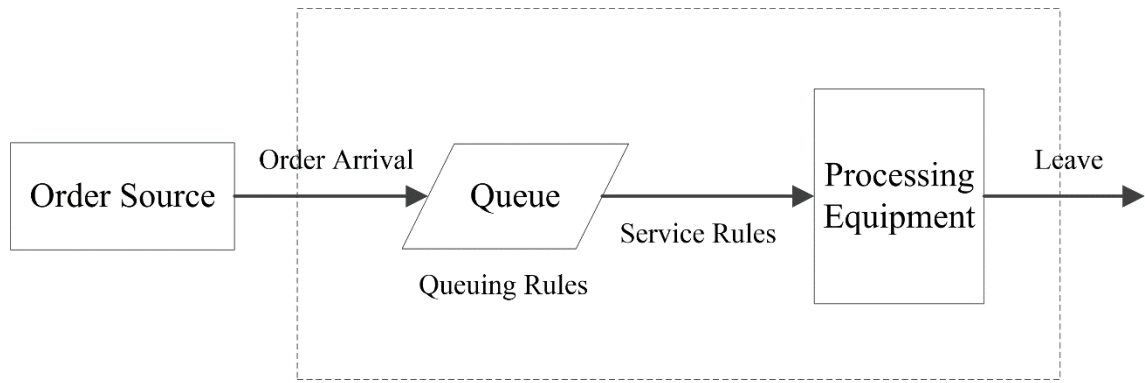

Supplement: S2 Fig — (PDF) [file pone.0261771.s002.pdf]

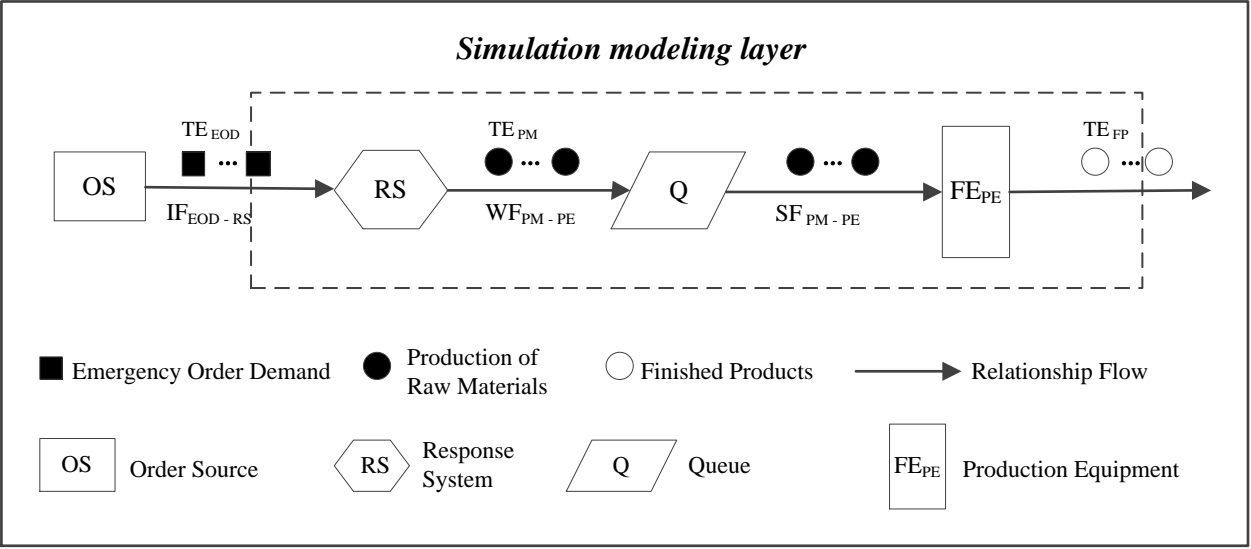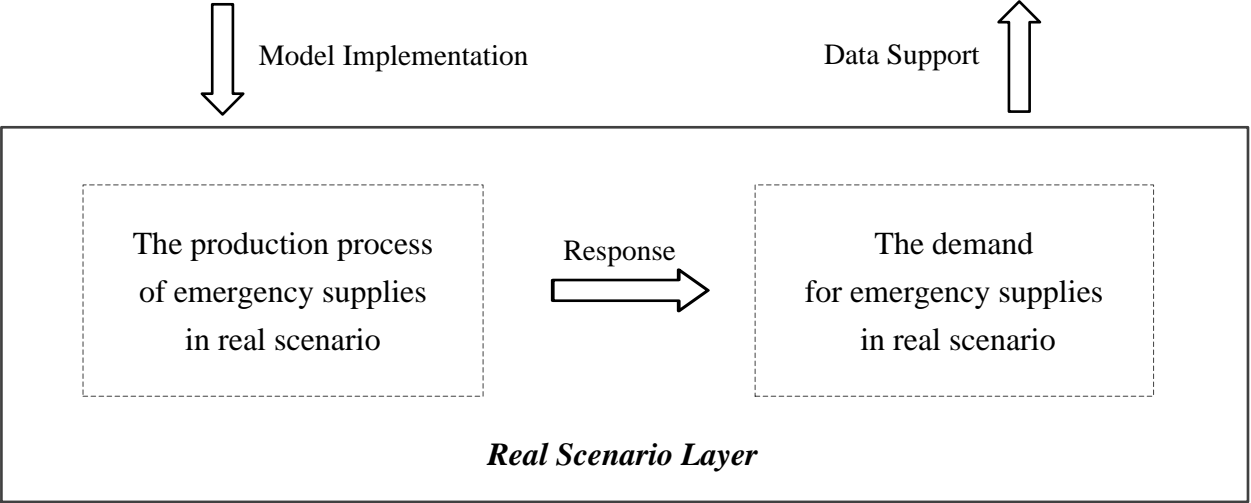

Supplement: S3 Fig — (PDF) [file pone.0261771.s003.pdf]

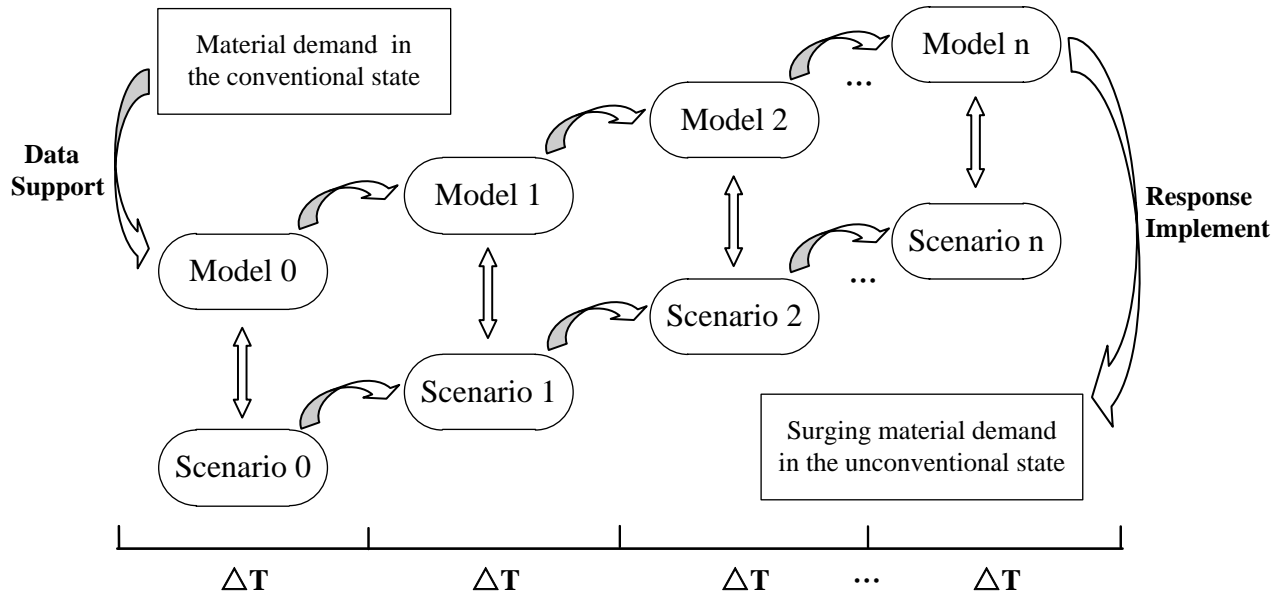

Supplement: S4 Fig — (PDF) [file pone.0261771.s004.pdf]

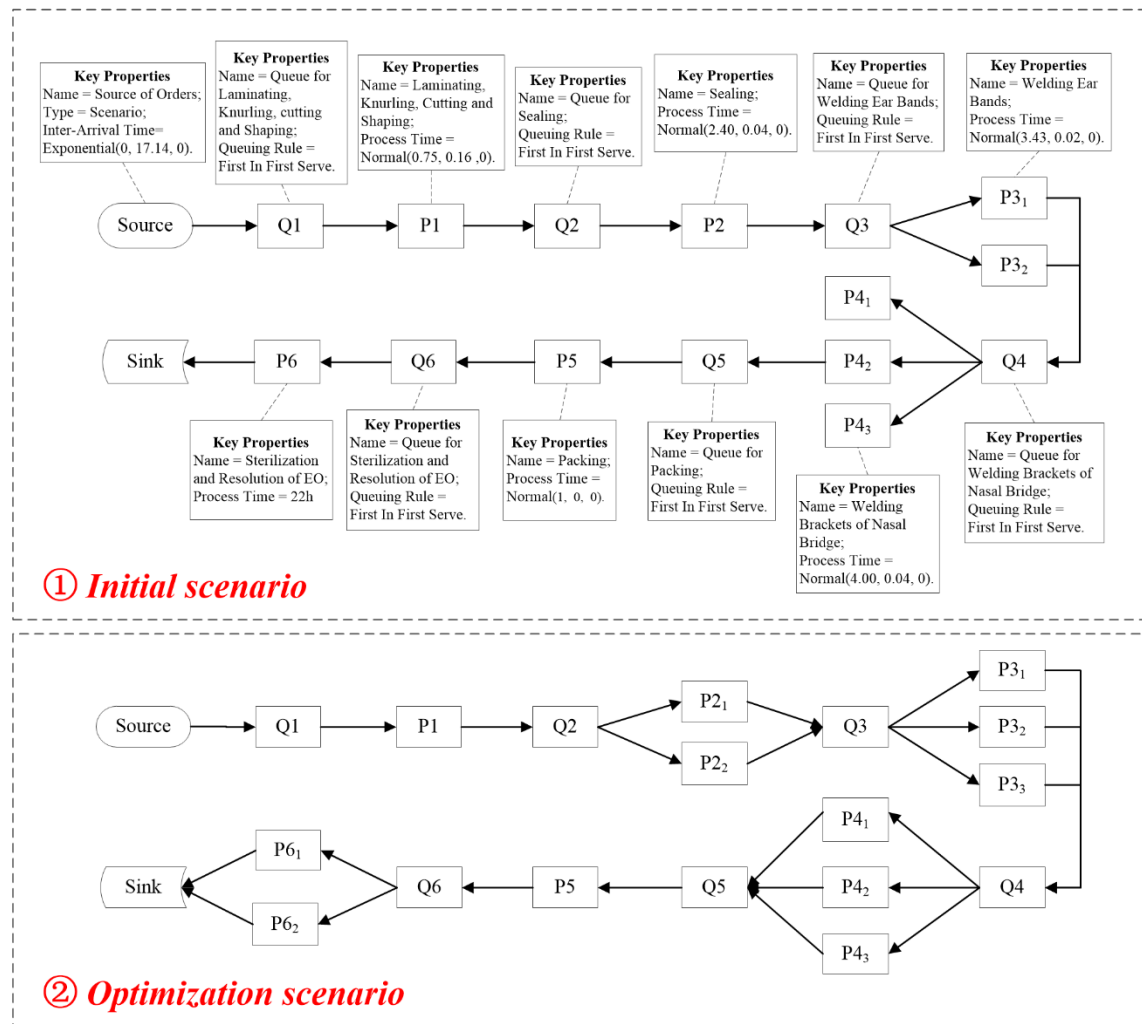

Supplement: S5 Fig — (PDF) [file pone.0261771.s005.pdf]

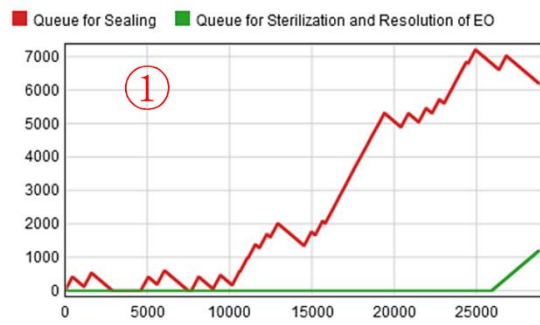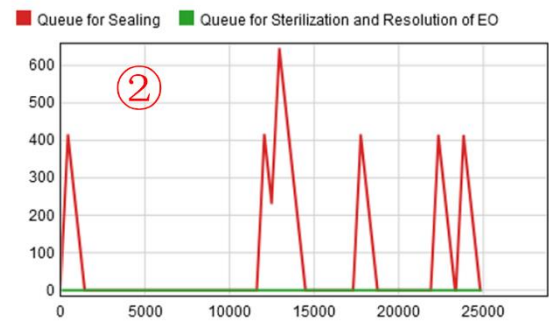

Supplement: S7 Fig — Scenario 0 (left), Model 0 (right) Staging Area “Time-Capacity”. (PDF) [file pone.0261771.s007.pdf]

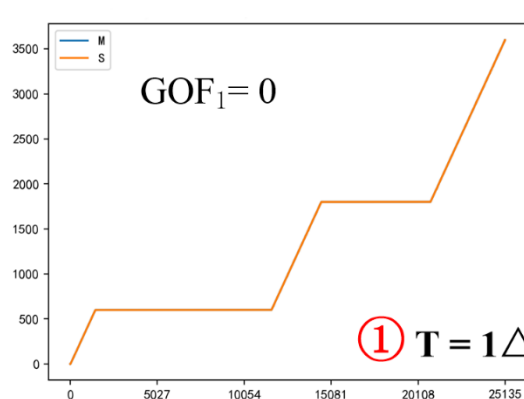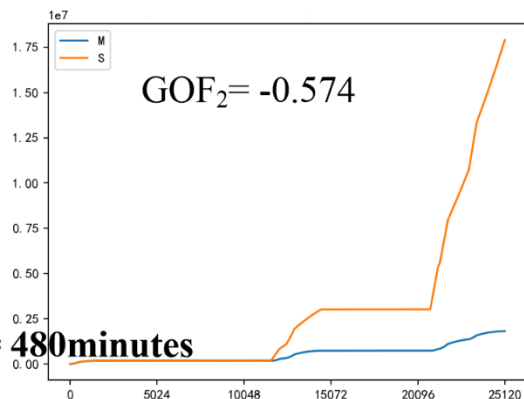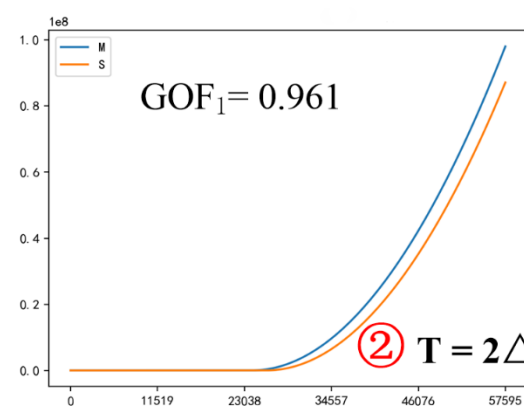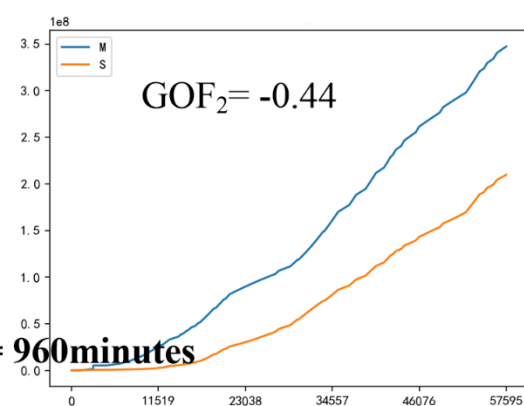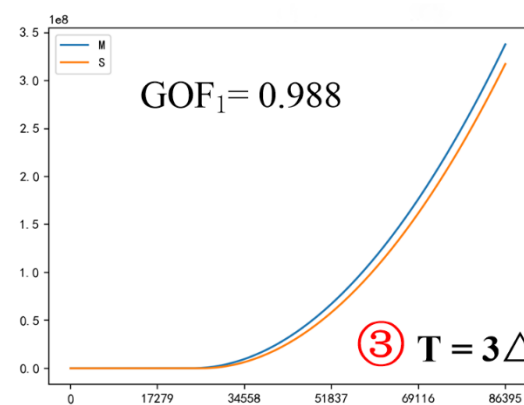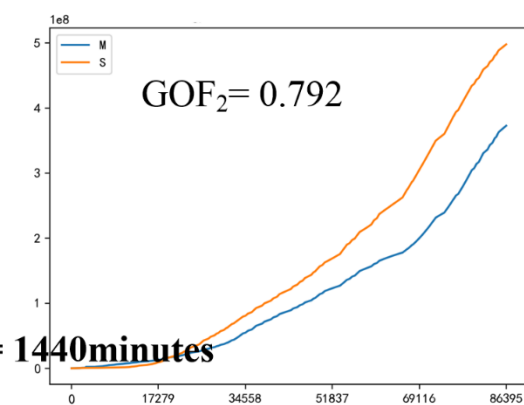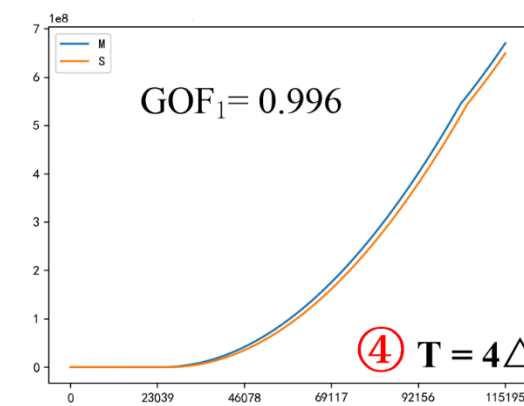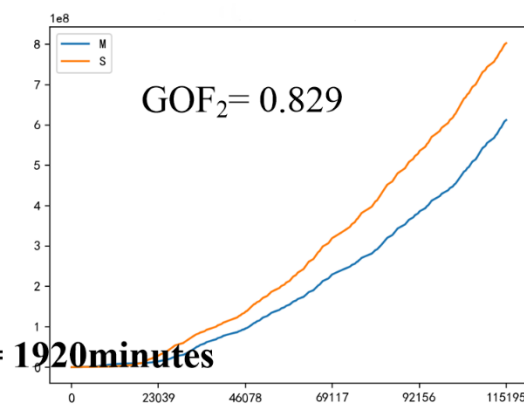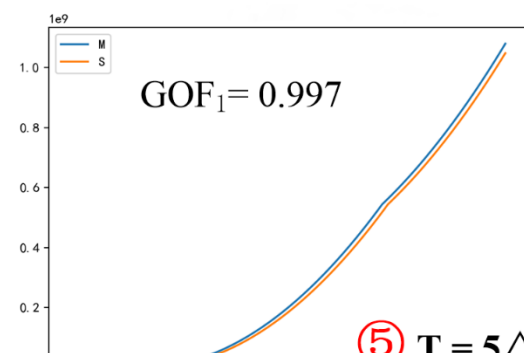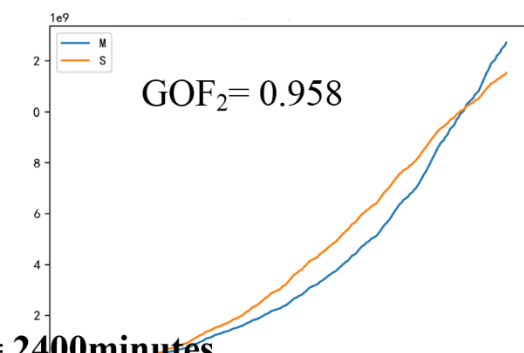

Supplement: S8 Fig — (PDF) [file pone.0261771.s008.pdf]

### *Before model optimization*

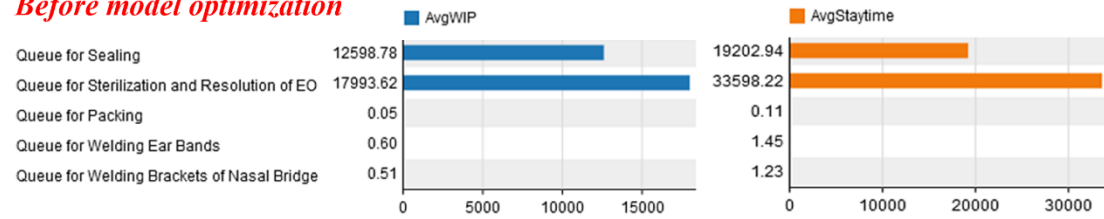

### *After model optimization*

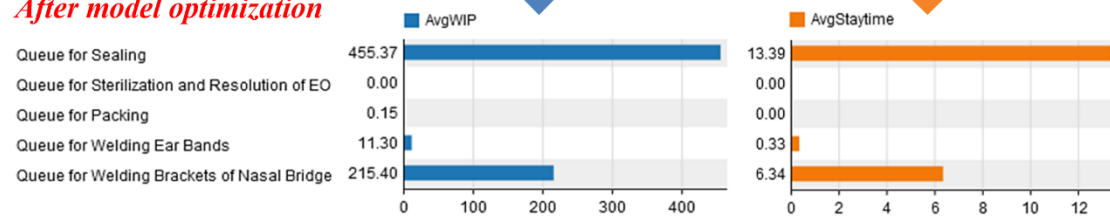

Supplement: S9 Fig — (PDF) [file pone.0261771.s009.pdf]
