# Supplementary material for: Parallel simulation and optimization framework of supplies production processes for unconventional emergencies
Source: PLoS One. 2022 Jan 13;17(1):e0261771. doi: 10.1371/journal.pone.0261771 (PMC8758009; doi:10.1371/journal.pone.0261771)
Supplement: S6 Fig — (PDF) [file pone.0261771.s006.pdf]

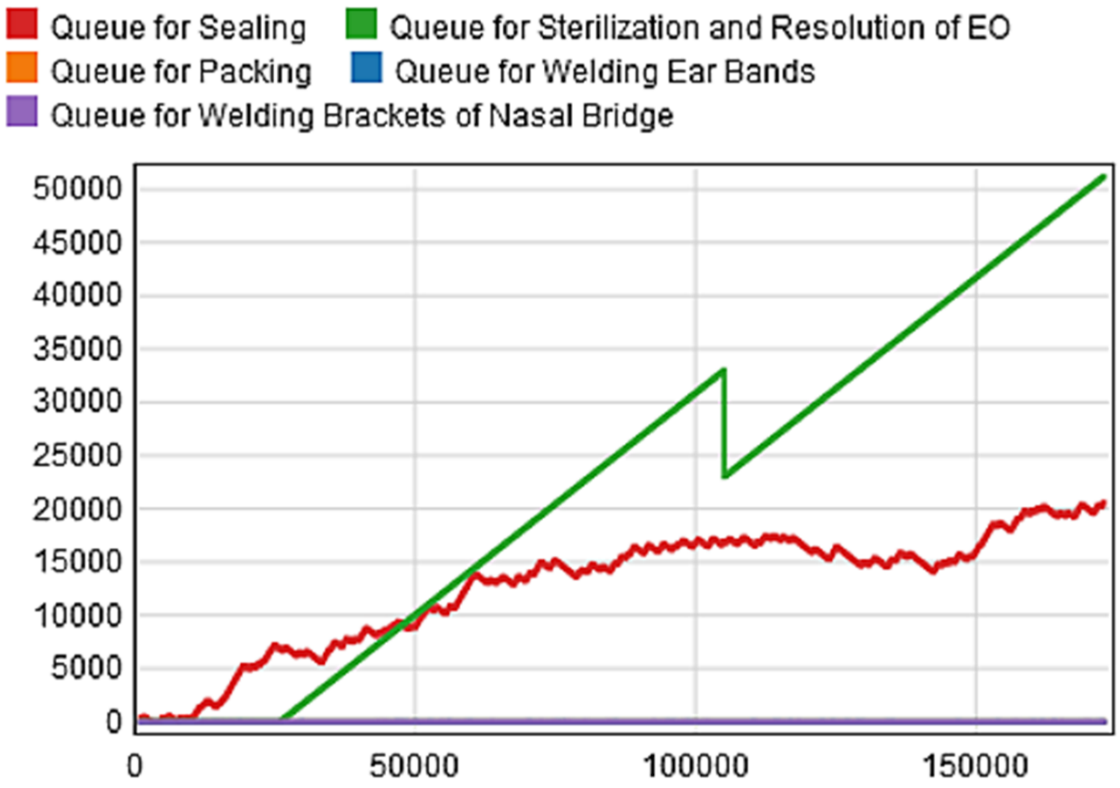

Queue for Sealing
Queue for Sterilization and Resolution of EO
Queue for Packing
Queue for Welding Ear Bands
Queue for Welding Brackets of Nasal Bridge
50000
45000
40000
35000
30000
25000
20000
15000
10000
5000
0
0
50000
100000
150000
