# Supplementary material for: Parallel simulation and optimization framework of supplies production processes for unconventional emergencies
Source: PLoS One. 2022 Jan 13;17(1):e0261771. doi: 10.1371/journal.pone.0261771 (PMC8758009; doi:10.1371/journal.pone.0261771)
Supplement: S10 Fig — (PDF) [file pone.0261771.s010.pdf]

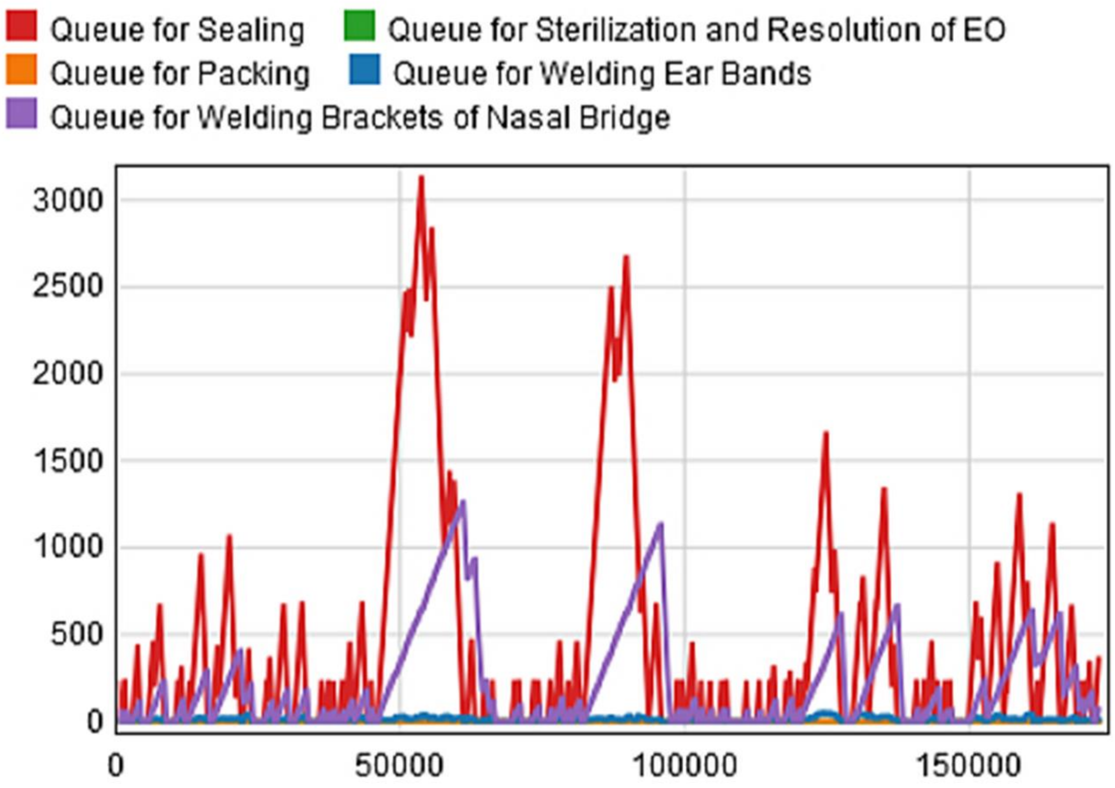

Queue for Sealing
Queue for Sterilization and Resolution of EO
Queue for Packing
Queue for Welding Ear Bands
Queue for Welding Brackets of Nasal Bridge
3000
2500
2000
1500
1000
500
0
0
50000
100000
150000
