# Supplementary material for: Parallel simulation and optimization framework of supplies production processes for unconventional emergencies
Source: PLoS One. 2022 Jan 13;17(1):e0261771. doi: 10.1371/journal.pone.0261771 (PMC8758009; doi:10.1371/journal.pone.0261771)
Supplement: S1 Table — (PDF) [file pone.0261771.s011.pdf]

**Simulation Model Elements**	**Real Scenario Elements**	**Related Elements in FlexSim**
Fixed Entity (FE)	Production Equipment	Fixed Resources: Processor, Combiner, Separator, etc.
Temporary Entity (TE)	Emergency Order Demand; Production of Raw Materials	Flow Items: Box, etc.
Relationship Flow (RF)		Port Connection
