# Supplementary material for: Parallel simulation and optimization framework of supplies production processes for unconventional emergencies
Source: PLoS One. 2022 Jan 13;17(1):e0261771. doi: 10.1371/journal.pone.0261771 (PMC8758009; doi:10.1371/journal.pone.0261771)
Supplement: S2 Table — (PDF) [file pone.0261771.s012.pdf]

**Process**	**Processing Time**
Laminating, Knurling, Cutting and Shaping	Normal (0.75, 0.16,0) s
Sealing	Normal (2.40,0.04,0) s
Welding Ear Bands	Normal (3.43, 0.02,0) s
Welding Brackets of Nasal Bridge	Normal (4.00, 0.04,0) s
Packing	Normal (1.00,0.00,0) s
Sterilization and Resolution of Ethylene Oxide	22 hours per batch
